# Enhancing the growth and yield of the common bean cultivar ’Nebraska’ under high temperature conditions by combining different magnesium levels with arginine, glycine, and melatonin

**DOI:** 10.1186/s12870-025-07088-3

**Published:** 2025-08-29

**Authors:** Sary H. Brengi, Ibrahim A. Abouelsaad, Ahmed A. Khadr, Mohamed Abdelghany

**Affiliations:** 1 Department of Horticulture, Faculty of Agriculture, Damanhour University, Damanhour, Beheira 22516 Egypt; 2 Faculty of Desert Agriculture, King Salman International University, Ras Sedr, 46618 Egypt; 3 Crop Science Department, Faculty of Agriculture, Damanhour University, Damanhour, 22516 Egypt

**Keywords:** Heat stress, Mature pods and seeds parameters, Vegetative characteristics

## Abstract

**Background:**

One of the most widely consumed legumes worldwide is the common bean. Abiotic stress factors such as heat stress significantly reduce crop productivity, and climate change models predict rising temperatures in many agricultural regions. In the 2021 and 2022 seasons, two field trials were conducted in the Wadi El Natrun Region, El-Behera Governorate, Egypt. A combined split-plot of the data was statistically evaluated using a Randomized Complete Block Design (RCBD). The following seven treatments were tested in this study: arginine at 1.25 and 2.5 mM, glycine at 12.5 and 25 mM, melatonin at 50, 100 µM, and control (distilled water). Magnesium fertilization treatments were added to the irrigation water at varying levels (0, 9.52, 19.05 and 28.57 kg/ha^−1^). Measurements of the physical and chemical characteristics, mature pods and seeds parameters, and vegetative features were made. This study aimed to ascertain whether common bean plants grown under natural field conditions in early April, thus exposes the plants to increasingly higher temperatures during critical reproductive stages towards late spring and early summer, particularly between May and July, when average daily temperatures in Egypt tend to exceed optimum values for common bean growth. This could benefit from the application of arginine, glycine, and melatonin to improve their development and yield, taking into account varying magnesium levels.

**Results:**

Melatonin at 100 µM produced the highest values across all measured characteristics, while the control treatment consistently showed the lowest values across all magnesium levels. Regarding magnesium levels, the highest values for all traits were observed at 28.57 kg ha⁻^1^, whereas the lowest values were recorded at 0 kg ha⁻^1^. There was a significant interaction between foliar application treatments and magnesium levels for plant height, potassium, phosphorus, and magnesium content. For each trait with a significant interaction, melatonin at 100 µM combined with magnesium at 28.57 kg ha⁻^1^ resulted in the highest values, whereas the control treatment with 0 kg ha⁻^1^ magnesium yielded the lowest values. Melatonin 100 µM under Magnesium level (28.57 kg ha⁻^1^) increased plant height to 65.46 cm, chlorophyll content to 43.41 SPAD units, and seed yield per plant to 26.4 g from 18.1 g in the control. Protein content was increased from 22.54% in the control to 23.98%. Mg content in seeds was up to 0.84% under this treatment as against 0.52% in the control.

**Conclusions:**

Results show that melatonin at 100 µM, combined with magnesium at 28.57 kg ha⁻^1^ produced the most favorable outcomes. This suggests a synergistic effect between melatonin and magnesium in enhancing the studied traits. These findings may also contribute to the future development of productive common bean breeding strategies.

## Introduction

The common bean (*Phaseolus vulgaris* L.) is a seasonal leguminous plant member of the *Leguminosae* family. Its production is more than twice as great as the next most significant grain legume, making it the most important grain legume for human consumption [1]. The common bean is one of the most significant protein sources [2]. It is abundant in dietary fiber, minerals, especially zinc and iron [3], and vitamins [4]. Since 1990, the area harvested of common beans has increased by 36%, but the production of dry beans has increased by roughly 60% [5]. It is extensively grown across numerous nations, with an approximate harvested area of 35.92 million hectares, yielding approximately 27.72 million tons of product [6]. However, various climate models indicate that this cultivated area could shrink by 50% by 2050 due to rising temperatures [7, 8].

In Egypt, the common bean is considered one of the most significant legume crops due to its high nutritional value [9]. It is grown for both local consumption and export, with about 36.7 thousand hectares harvested, yielding 144.8 thousand tons. The high temperatures in Egypt from April to August increasingly hinder common bean development and productivity [3]. In the context of global warming, understanding heat stress impacts on plant productivity and quality is essential for developing climate adaptation strategies [10, 11]. Though earlier research has determined the nutritional value of the common bean and its sensitivity to heat, few studies have examined combined mitigation approaches involving melatonin, glycine, arginine, and magnesium. Exogenous melatonin, glycine, and arginine were used in this study as stress-relieving substances: melatonin is an effective antioxidant and stress-inducible gene modulator; glycine enhances osmo-protection and stabilizes cellular structures; and arginine promotes nitric oxide production to protect against antioxidants and ensure cell homeostasis under heat shock [12].

One of the most significant abiotic stresses affecting the production of numerous species of plants is high temperature [13]. It reduces crop yield and threatens nutrition security, especially under ongoing climate change [14]. Plants undergo a series of physiological and biochemical reactions when exposed to heat, which can have detrimental effects, like oxidative stress [15]. High temperatures cause pollen viability to decline, anther dehiscence to occur, flowers, buds, and pods to abort, and decrease seed size and filling, which will significantly lower yield [14]. It has been noted that temperatures greater than 20 °C at night or more than 30 °C throughout the day reduce seed yield [16]. To live in a comparatively warm and unpredictable climate, Nebraskan bean genotypes most likely possess particular adaptations. These adaptations may include physiological characteristics such as drought resistance, heat tolerance, or effective photosynthesis at elevated temperatures. Higher-temperature growing zones may cause beans to evolve defenses against heat stress, conserve water, and adjust their growth cycle to suit the local climate [17]. Nebraska beans are therefore anticipated to be more temperature-tolerant [18].

Magnesium (Mg) is a crucial component of crops, whose deficiency impacts crop photosynthesis and carbohydrate partitioning [19], decreases the output of agriculture and the sustainability, and has detrimental long-term effects on the health of plants [20]. Additionally, phloem loading and amino acid allocation in plants are decreased by Mg deficiency [21]. Regretfully, crops usually exhibit clear signs of Mg deficiency, particularly during their crucial developmental stage when fast carbohydrate accumulation occurs, grown on acidic soils that are widely found worldwide [21]. For the health of plants and people, agricultural products'Mg contents must be kept within a reasonably sufficient range [22].

It has been widely documented that amino acids have a bio-stimulating influence on plant development and nutrient absorption improvement [23, 24]. Because of their nature, low rates of application, and increased effectiveness, amino acids are completely safe for the soil and environment [13]. One of the most prevalent free amino acids in soil, glycine, is the most basic amino acid found in nature [12]. Glycine is one of the amino acids used most frequently in plant nourishment. In comparison to other amino acids, plants absorb it more quickly [25]. It is frequently employed in the synthesis of numerous amino-chelate fertilizers, also known as amino acid-chelated nutrients [26]. The number of foliar or soil applications, species of plants, development stage, climate, and, most importantly, the applied level all influence how effective the applied amino acid (glycine) is [25]. L-arginine is the most operationally variable amino acid in plants and a precursor for the creation of polyamines and nitric oxide, according to certain studies [27]. In crops, polyamines and their precursor arginine have been identified as essential regulators of numerous processes, including growth, physiology, and plant reaction to diverse stressors [14]. Additionally, it has been observed that arginine, either endogenous or exogenous, contributes to the response to stress in plants [28].

N-acetyl-5-methoxy tryptamine, or melatonin, was identified in 1995 as a significant bioactive substance in vascular plants [11]. It can be found in the tissues of different plants, including the seeds, roots and leaves [29]. It was once thought to be a potent antioxidant with a variety of advantageous functions during different phases of plant development and growth [30] For instance, germination [12], photosynthesis [15], and the senescence of leaves [31]. It is additionally a plant hormone that plays a significant part in promoting plant development and regulation [10].

It is anticipated that global temperatures will rise as a result of the ongoing global warming phenomenon [32]. Consequently, to develop new tactics for adaptation to the scenario of climate change, it is crucial to investigate comprehensively how heat stress may impact plant production as well as quality [33]. From April to August, the prevailing weather conditions in Egypt are not suitable for the growth and production of high-quality common beans, whether in the open fields.

Despite this information, there remains a knowledge gap on how these compounds combine to enhance common bean tolerance to extended heat. The study investigated whether exogenous application of arginine, glycine, melatonin, and varying concentrations of Mg can reverse the negative effect of heat stress on common bean growth and yield. The study bridges this gap, founded on the hypothesis that these agents combined increase physiological activity, growth, and yield under high-temperature stress. Therefore, the objectives were to (1) evaluate the individual and combined effects of arginine, glycine, melatonin, and Mg on the growth and yield of common beans under heat stress, (2) investigate the physiological mechanisms of improved heat tolerance, and (3) identify the optimal combinations of treatments for common bean productivity improvement in warm environments.

## Materials and methods

### Plant materials and field valuation

The Nebraska common bean cultivar's seeds were obtained from the Horticulture Institute, Agriculture Research Centre, Egypt. Two field experiments were conducted during the 2021 and 2022 seasons in Wadi El Natrun Region, El-Behera Governorate, Egypt (location: latitude 30.42, longitude 30.34). To recognize the soil physico-chemical characteristics of the experimental site before the initiation of planting, soil samples at different places of the field at a depth of 25 cm were collected and analyzed according to the methods reported [34]. The results of soil physico-chemical analyses are shown in Table 1 and the weather conditions that occurred at the experiment site during the experiment are shown in Table 2.

**Table 1:** Some physical and chemical characteristics of the experimental sites during the 2021 and 2022 seasons, including texture, pH, electrical conductivity, organic matter, and essential nutrients

							%			(ppm)		(mg L^−1^)				
Season	Sand (%)	Silt (%)	Caly (%)	Textural class	pH	EC (dS m^−1^)	O.M	CaCO_3_	N	P	K	Na	Ca	Mg	Cl	SO_4_
2021	85.1	9.3	5.6	Sandy	7.79	1.18	0.39	1.71	0.09	21	56	4.27	8.11	6.13	6.21	6.21
2022	86.2	7.5	5.3	Sandy	7.91	1.09	0.35	1.64	0.12	27	64	4.41	7.60	5.86	6.08	6.17

EC = electrical conductivity and O.M. = organic matter

**Table 2:** The weather conditions prevailing in a location during the 2021 and 2022 seasons at two meters, showing maximum and minimum temperatures, humidity, dew point, and wet bulb temperature. Data is retrieved from: Power.larc.nasa.gov/data-access-viewer

	Temperature Maximum (C)		Temperature Minimum ©		Relative Humidity (%)		Specific Humidity (g/kg)		Dew/Frost Point (C)		Wet Bulb Temperature (C)	
	2021	2022	2021	2022	2021	2022	2021	2022	2021	2022	2021	2022
APR	40.89	40.83	7.4	8.99	53.88	51.83	6.71	6.9	7.8	8.02	14.38	14.73
MAY	42.72	42.31	15.07	12.73	44.12	47.81	7.87	7.87	10	9.95	17.73	17.37
JUN	41.53	45.35	16.26	18.55	47.62	48.81	9.64	10.5	13.08	14.38	19.94	21.62
JUL	44.12	41.3	20.32	19.88	48.19	49.56	11.41	11.11	15.66	15.04	22.48	22.39

NASA/POWER CERES/MERRA2 Native Resolution Monthly and Annual (Location: Latitude 30.42 Longitude 30.34). Parameter(s) at 2 m

### Soil preparation and seed sowing

The experimental site was properly plowed and leveled, and the plots were prepared according to the layout plan. Common bean seeds (cv. Nebraska) were semi-automatically sown using a planting machine. Planting was done in three rows, each 0.5 m wide, located at the back of the terrace. Three drip irrigation lines were installed, with a spacing of 10 cm between seeds along each line. Planting was carried out on 1 and 2 April in 2021 and 2022, respectively. The land area consisted of three terraces, each 1.5 m wide, resulting in a total width of 4.5 m. The length of the terraces was 40 m, which corresponded to the length of the drip lines, giving a total area of 180 square metres (4.5 m × 40 m). Adjacent treatments were separated by one unplanted row to prevent spray drift. Before sowing, basal dressing with nitrogen, phosphorus, and potassium fertilizers at the prescribed levels for commercial common bean production was applied. Specifically, 45 kg N per feddan of nitrogen and 31 kg P₂O₅ per feddan of phosphorus, and 48 kg K₂O per feddan of potassium were applied. In addition, seeds of the common bean cultivar'Nebraska'were inoculated with *Rhizobium leguminosarum* bv. *phaseoli* strain LCS0306, a highly efficient nitrogen-fixing strain compatible with common bean. Crop growth lasted from April to August in both seasons, with the crops reaching physiological maturity approximately 100 days after sowing.

### Treatment application and agro-management practices

Magnesium fertilization (Magnesium nitrate) treatments at different levels (0, 9.52, 19.05 and 28.57 kg/ha^−1^) were added to the irrigation water and the following seven treatments were tested in this study: arginine (1.25 and 2.5 mM, Sigma-Aldrich, purity 98%), glycine (12.5 and 25 mM, Sigma-Aldrich, purity 99%), melatonin (50 and 100 µM, Sigma-Aldrich, purity 98%), and control (distilled water). At a suitable wind speed to minimize spray drift, treatments were applied as foliar spray until run-off three times: 15, 30, and 45 days after seed sowing. All the other agro-management practices, such as cultivation, irrigation, nutrition, and pest control, were performed whenever it was necessary and as recommended for the commercial production of common beans.

The concentrations of Mg, arginine, glycine, and melatonin employed were determined from previous research and preliminary experiments under similar environmental conditions. The levels of Mg were employed to create a range from deficiency to adequacy to span the scope of the physiological response of the plant. Levels of arginine, glycine, and melatonin were selected to establish their efficacy in reducing heat stress at low and high rates of application and to achieve a complete understanding of their potential effects.

### Plant morphological characteristics

#### Vegetative characters

Plant samples from five random plants were collected from each experimental unit, and 60 days of seed sowing were used to measure the morphological characters. Plant height (cm) was measured with the help of a measuring scale from the soil surface to the tip of the main stem. Samples were washed with tap water, then washed with distilled water three times, and dried in a forced-air oven at 70 °C till constant weight. The number of leaves per plant was counted. Plant leaves area (cm^2^/plant) was mathematically calculated using the relationship between the dry weight of leaves per plant on one side and the dry weight and area of 20 discs taken from fresh leaves by a borer with a known diameter on the other side and the dry weight leaves per plant as follows:

Leaf area (cm^2^) = leaves dry weight (g) × 20 discs area (cm.^2^)/20 discs dry weight (g) [35]

#### Mature pods parameters

In each experimental unit, green pods were picked throughout the entire harvesting period, and the following data were recorded: number of green pods per plant, average green pod weight (g/pod) and average dry pod weight (g/pod).

### Dry seed yield and its components

In each experimental unit, plants in the first row were left to grow till pods approached the dry stage (12–14% seed moisture content), and the following measurements were performed: Number of seeds per pod, seed yield per plant, seed yield per feddan and weight of 100 seeds.

### Chemical constituents

Leaves and seed samples were ground in a Willy mill to pass a 30-mesh screen. Weights of 0.2 g of dried fine powder from the samples were digested according to [36], and the following determinations were achieved: Total nitrogen (%), determined using the Micro Kjeldahel apparatus (VELP Scientifica UDK 159) as defined by Chapman and Pratt (1965)[37]. The other minerals were analyzed after first drying at 550 °C in a Muffle furnace and being dissolved in deionized water to a standard volume. Potassium was determined by flame photometry (Jenway PFP7, UK), while phosphorus was determined by the vanadomolybdate method [38]. Crude protein% was calculated as N% % × 6.25. The rest of the minerals (Ca, Mg, Zn, Fe, and Mn) were determined using an atomic absorption spectrophotometer (PerkinElmer AAnalyst 400) [39]. The third fully grown leaf from each plant's apex was used to measure the amount of chlorophyll. To reduce diurnal variability, readings were obtained during flowering and at regular intervals between 8:00 and 10:00 AM. A SPAD-502 chlorophyll meter (SPAD unit) (Minolta Camera Co., Ramsey, NJ) was used to measure the amount of chlorophyll [40].

### Statistical analysis

Treatments were distributed in completely randomized block trials using three replications. Each experimental unit was planned to cover an area of 180 m^2^, including 12 rows of 40 m in length and 4.50 m in width, separated by a bar row. A Randomized Complete Block Design (RCBD) was used to statistically evaluate the data as a combined split plot. Foliar applications of arginine, glycine, control, and melatonin were the subplots, while the Mg fertilization treatments were the main plot. The homogeneity of each trait was ascertained using the Bartlett test [41]. LSD was used to evaluate treatment mean differences at a significance level of 0.01 probability [42]. To find the correlation coefficient between different traits, R v3.5.1 was utilized.

## Results

### Vegetative characters

**Fig. 1:**
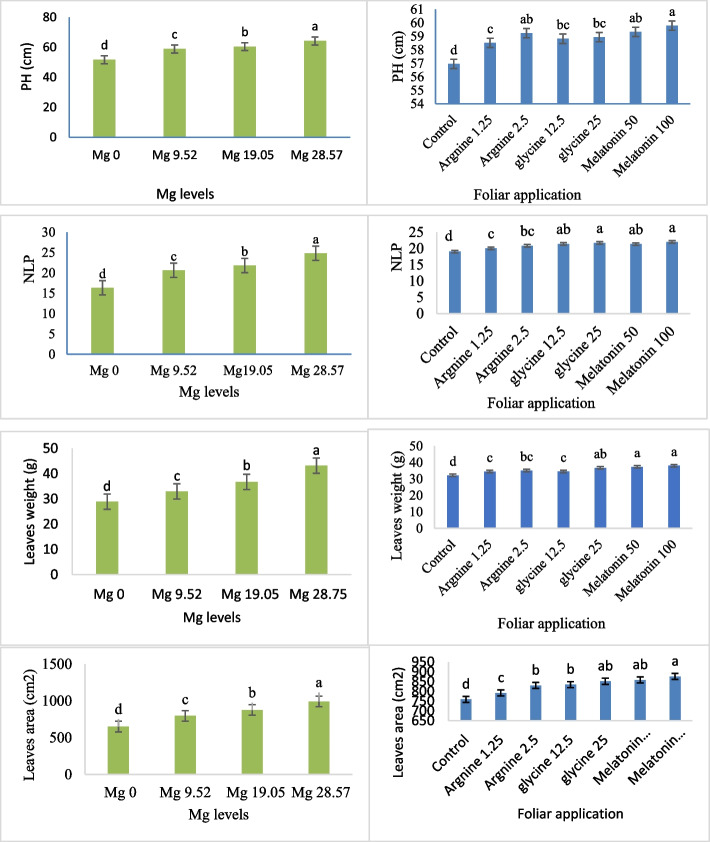
Effects of foliar arginine and glycine applications, magnesium fertigation levels, control, and melatonin treatment on plant height (PH, cm), number of leaves per plant (NLP), leaf weight (g), and leaf area (cm2). At *p* < 0.01, bars with distinct letters are considered statistically significant. Standard errors of the mean are shown by error bars

Under the Mg fertigation levels and foliar application of arginine, glycine, control, and melatonin, the plant's height, number of leaves per plant, leaf weight, and plant leaf area features were measured. The impacts of foliar spray and fertigation Mg levels on all vegetative features were extremely significant (*P* < 0.01), according to an ANOVA analysis (Table 3). All vegetative variables, except plant height, were not significantly impacted by the interactions between Mg fertigation levels and foliar application (Table 3). Among magnesium treatments, melatonin 100 µM produced the highest average plant height (59.8 cm), number of leaves per plant (22.04), leaf weight (38.00 g), and leaf area (875.76 cm^2^). But in terms of plant height (56.95 cm), number of leaves per plant (19.04), leaf weight (32.15 g), and leaf area (758.49 cm^2^), the control (distilled water) was the lowest. (Fig. 1). In comparison to the Mg concentration (0 kg ha⁻^1^), which produced the following results (plant height 51.66 cm, number of leaves per plant 16.35, leaves weight 28.91 g, and plant leaves area 650.56 cm^2^), the Mg treatment (28.57 kg ha⁻^1^) had the highest levels of all the traits (plant height, number of leaves per plant, leaves weight, and plant leaves area) as follows: 64.23 cm, 24.83, 43.2 g, and 991.08 cm^2^) (Fig. 1).

**Table 3:** ANOVA (analysis of variance) summary table for the vegetative traits of common bean plants across two seasons, evaluating the effects of foliar treatments and Mg fertigation over the two years

Source of variation	Replication	Magnesium levels (A)	Foliar application treatments (B)	A*B
D.F	2	3	6	18
PH (cm)	1.210	582.308^**^	10.029**	2.773^**^
Number of leaves per plant	0.083	259.313^**^	13.208^**^	0.882^ ns^
Leaves weight (g)	8.493	773.904**	48.677**	2.171^ ns^
Leaves Area (cm^2^/Pl)	612.78	430,220.32**	19,850.758**	572.166^ ns^

^**^ significance at 0.01 level

^ns^: Non-significant

**Fig. 2:**
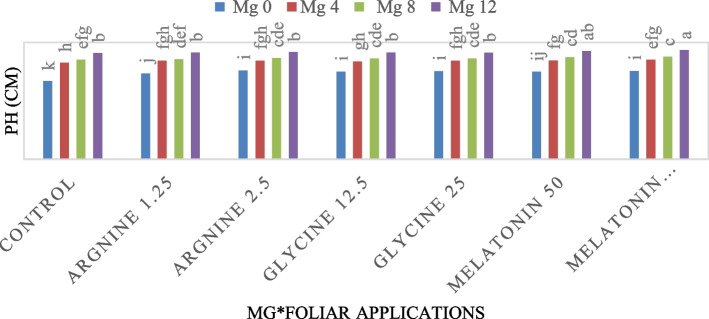
Effect of Mg fertigation levels and the interplay of arginine, glycine, control, and melatonin administration given foliarly on plant height (PH, cm). At *p* < 0.01, bars with distinct letters are considered statistically significant. Standard errors of the mean are shown by error bars

The highest plant height (65.46 cm) was obtained with melatonin 100 µM under magnesium level (28.57 kg ha⁻^1^), according to the interaction between Mg fertigation levels and foliar spray treatments. Conversely, the lowest plant height (46.78 cm) was obtained with the control treatment at Mg level (0 kg ha⁻^1^) (Fig. 2).

**Fig. 3:**
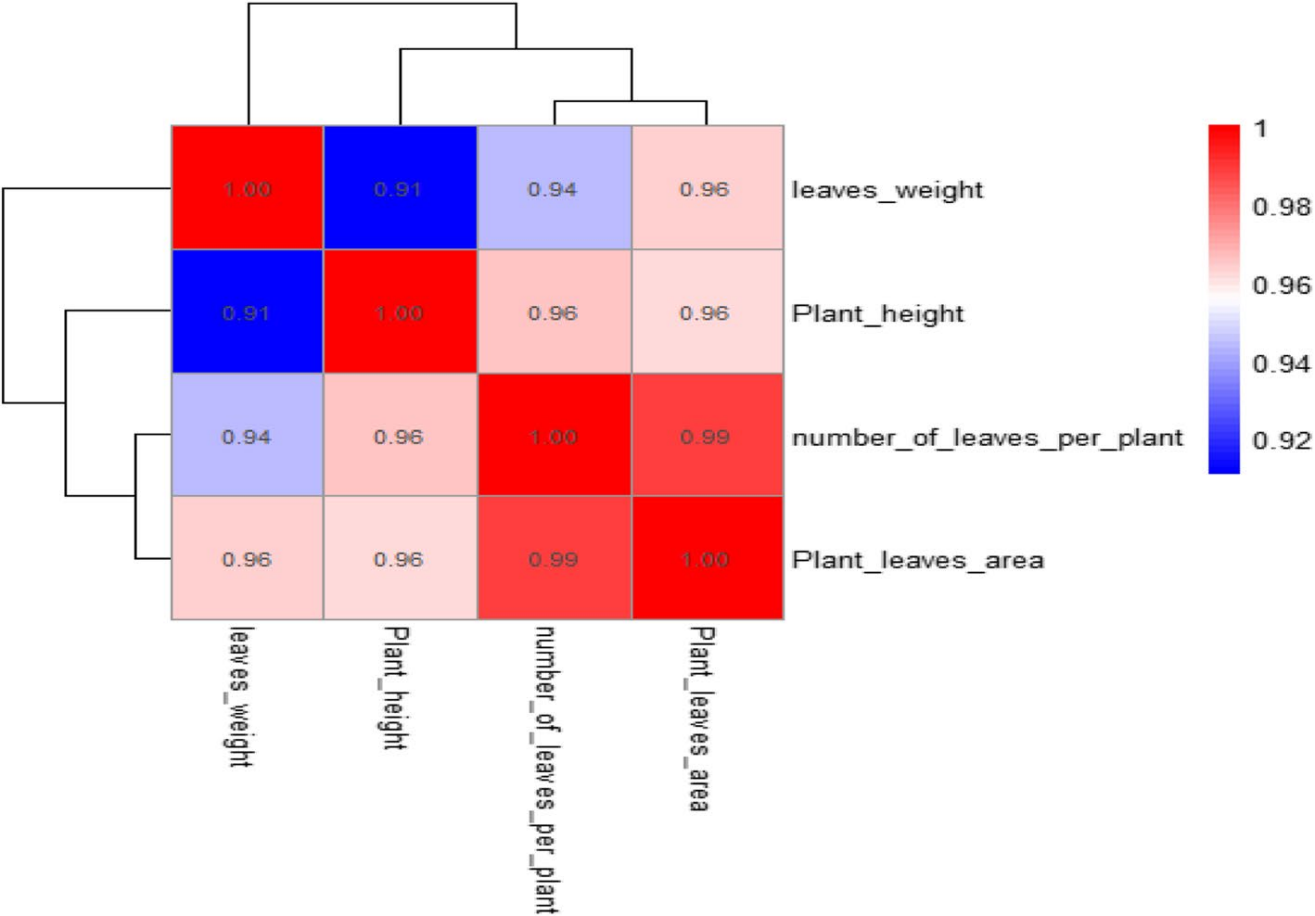
The Pearson heat map for plant height (PH, cm), number of leaves per plant (N. leaves/Pl), leaves weight (gm) and plant leaves area (cm^2^/plant) traits across the Mg levels and arginine, glycine and melatonin levels. The numbers in the figure refer to the correlation coefficients between the four traits. The correlation matrix displays significant relationships (*p* < 0.01). The color scale indicates the strength and direction of the correlation, with red representing strong positive correlations and blue indicating weaker correlations. The color intensity indicates the strength and direction of the relationship

Strong relationships between all vegetative qualities were shown by the correlation heatmap clustering (Fig. 3). The strongest link was found between the area of plant leaves and the number of leaves per plant (*r* = 0.99). Notably, there was a substantial correlation between plant height and the number of leaves per plant (*r* = 0.96), as well as between plant height and the area of plant leaves (r = 0.96) (Fig. 3). In a similar vein, there was a significant correlation (*r* = 0.96) between the weight of the leaves and the area of the plant leaves. Furthermore, the area of the leaves and the number of leaves per plant (0.96) were significantly correlated with plant height (*r* = 0.96). Additionally, the weight of the leaves and the number of leaves per plant were significantly correlated (*r* = 0.94), although the weight of the leaves and plant height were less significantly correlated (*r* = 0.91) (Fig. 3).

### Parameters of mature pods

**Fig. 4:**
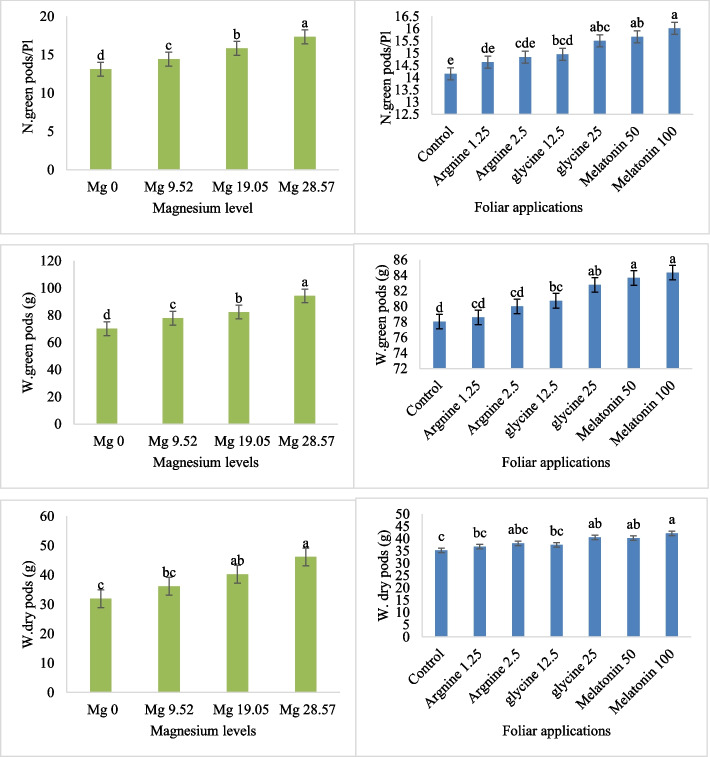
The effect of the Mg fertigation levels, foliar application of arginine, glycine, control and melatonin on the green pods’ number per plant (N. green pods/Pl), weight of dry pods (W. dry pods, g) and weight of green pods per plant (W. green pods, g). At *p* ≤ 0.01, bars with distinct letters are considered statistically significant. Standard errors of the mean are shown by error bars

According to the ANOVA analysis, the foliar spray treatments and Mg levels were highly significant (*P* < 0.01) for the mature pods'parameters (number of green pods per plant, the weight of dry pods, and the weight of green pods per plant) (Table 4). However, the interactions between the Mg levels and foliar spray treatments significantly impacted none of the features (Table 4). The three features (number of green pods per plant, weight of dried pods, and average green pod weight) showed that the Mg content was highest at 28.57 kg ha⁻^1^and lowest at 0 kg ha⁻^1^ (Fig. 4).

**Table 4:** Analysis of variance (ANOVA) summary table for characteristics of mature pods of common bean under different foliar spray treatments (arginine, glycine, melatonin, control) and Mg fertigation levels across two growing seasons

Source of variation	Replication	Magnesium levels (A)	Foliar application treatments (B)	A*B
D.F	2	3	6	18
Number of green pods/plant	0.085	67.065^**^	5.0366^**^	0.456^ ns^
Weight of green pods (g)	46.342	2140.292^**^	73.845^**^	8.794^ ns^
Weight of dry pods (g)	1.138	766.624**	70.468^**^	8.0799^ ns^

^**^: significance at 0.01 level

^ns^: Non-significant

Melatonin 100 µM showed the greatest values of the three features (number of green pods per plant, weight of dry pods, and weight of green pods per plant) (16.01, 42.16 g, 84.36 g), respectively (Fig. 4). The control (distilled water) exhibited the lowest values of the three features (number of green pods per plant, weight of dry pods, and weight of green pods per plant), measuring 14.15, 35.24 g, and 78.05 g, respectively (Fig. 4).

**Fig. 5:**
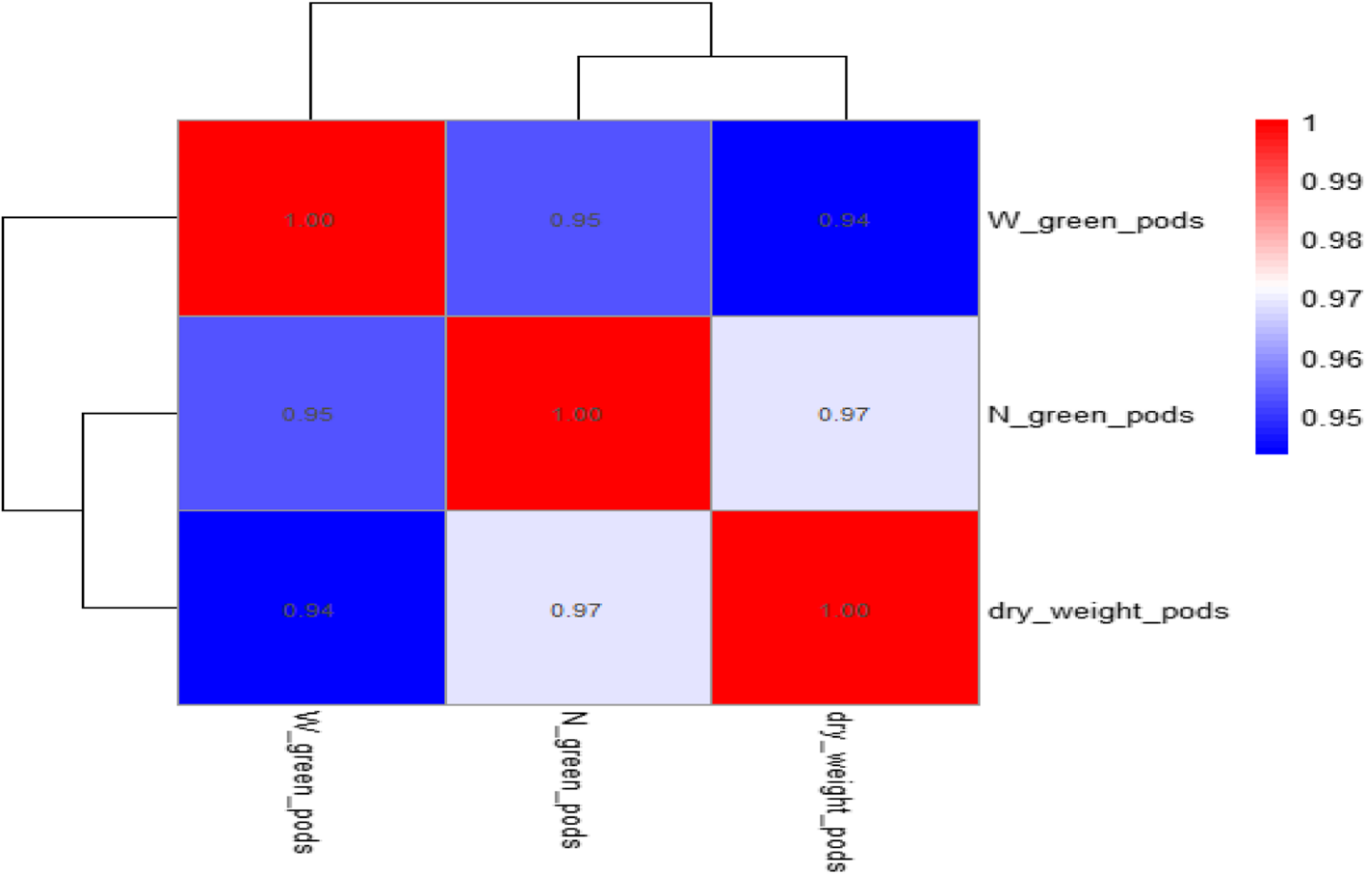
The Pearson’s association heatmap between the weight of green and dry pods and the number of green pods produced per plant (N. green pods/Pl), across the Mg levels and arginine, glycine, and melatonin levels. The figure's numbers represent the correlation coefficients between the three attributes. With blue colors denoting weaker correlations and red colors denoting strong positive correlations, the color scale shows the correlation's direction and intensity

The correlation matrix shows significant positive connections between the three pod attributes (number of green pods per plant, weight of green pods, and dry weight of pods). As one trait increases, the others usually do the same, according to the correlation coefficients, which range from 0.94 to 0.97. The strongest correlation (*r* = 0.97; *P* < 0.01) is seen between the dry weight of pods and the quantity of green pods per plant (Fig. 5).

### Dry seed yield and its components

**Fig. 6:**
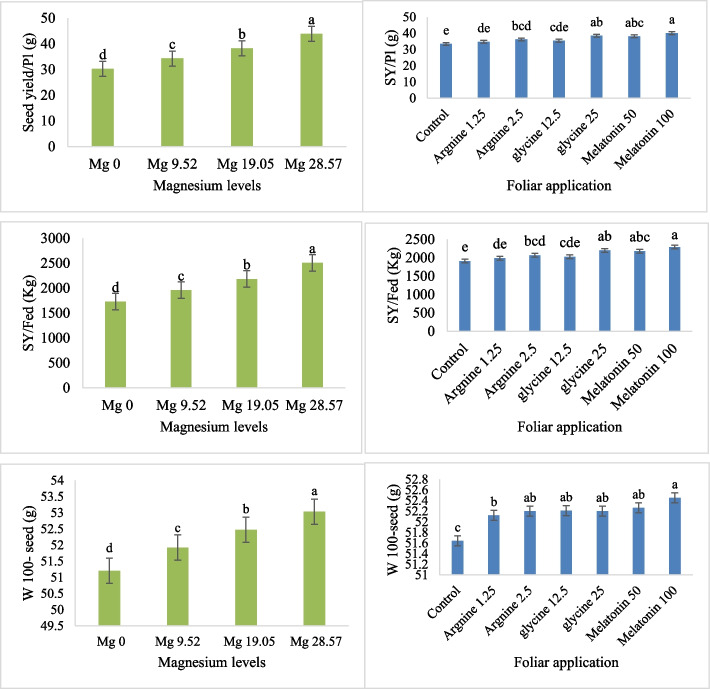
Effect of the Mg fertigation levels, foliar application of arginine, glycine, control and melatonin on the number of seeds per pod (N. seeds/pod), seed yield per plant (SY/Pl, g), seed yield per feddan (SY/Fed, Kg) and weight of 100-seeds (W. 100-seeds, g). At *p* < 0.01, bars with distinct letters are considered statistically significant. Standard errors of the mean are shown by error bars

The characteristics of dry seed yield and its components were examined, including the seed yield per plant, seed yield per feddan, the number of seeds per pod and the weight of 100 seeds. The ANOVA analysis revealed that, except for the number of seeds per pod, the effects of Mg levels and foliar spray treatments were very significant (*P* < 0.01) for every parameter (Table 5). The interactions between Mg levels and foliar spray treatments were not significant for all seed traits (Table 5). For every seed characteristic, the relationship between Mg levels and foliar spray treatments was negligible. Among all significant attributes across seaweed treatments, the melatonin level (100 µM) was the highest, whereas the control treatment had the lowest amount (Fig. 6). For all features, the maximum concentration of magnesium was at 28.57 kg ha⁻^1^, while the lowest concentration was at 0 kg ha⁻^1^ (Fig. 6).

**Table 5:** Analysis of variance summary table for common bean seed yield and its component parameters during two growing seasons with Mg fertigation and several foliar spray treatments (arginine, glycine, melatonin, and control)

Source of variation	Replication	Magnesium levels (A)	Foliar application treatments (B)	A*B
D.F	2	3	6	18
No.Seeds/pod	0.0386	0.289^ ns^	0.107^ ns^	0.067^ ns^
Seed yield/plant (g)	1.220	708.95^**^	65.749^**^	7.757^ ns^
Seed yield/Fed (Kg)	3978.706	2,314,531.5^**^	214,590.12^**^	25,330.705^ ns^
Weight of 100 seeds (g)	0.296	12.808**	0.734^**^	0.102^ ns^

^**^: significance at 0.01 level

^ns^: Non-significant

**Fig. 7:**
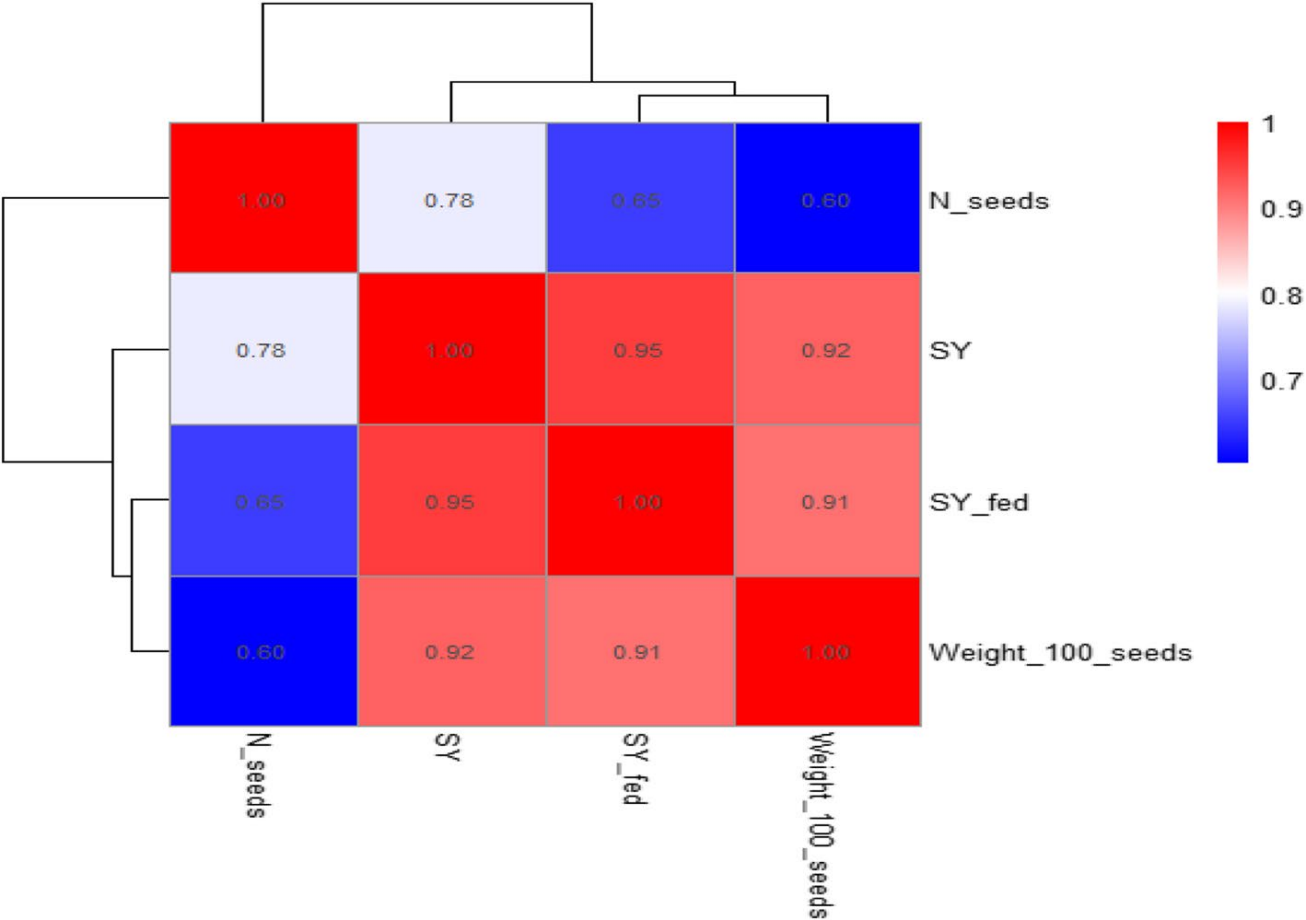
The relationship between seed yield per plant (SY/Pl), seed yield per feddan (SY/Fed), the number of seeds per pod (N. seeds/pod) and the weight of 100 seeds, as influenced by Mg, arginine, glycine, and melatonin levels. The numbers in the figure show the three qualities'correlation coefficients. The correlation's direction and intensity are indicated by the color scale; blue denotes lesser correlations while red denotes strong positive correlations

Significant correlations between the investigated seed yield and its related traits were found. Seed yield per plant and seed yield per feddan showed a very strong positive connection (*r* = 0.95) (Fig. 7). The weight of 100 seeds and seed output per plant were significantly positively correlated (*r* = 0.92). Likewise, there was a strong positive correlation (*r* = 0.91) between the weight of 100 seeds and the seed production per feddan. There was a relatively positive correlation between the number of seeds per plant and the seed production per plant (*r* = 0.78). The lowest positive association (*r* = 0.60) is found between the weight of 100 seeds and the quantity of seeds per plant (Fig. 7).

### Physical and chemical parameters

**Fig. 8:**
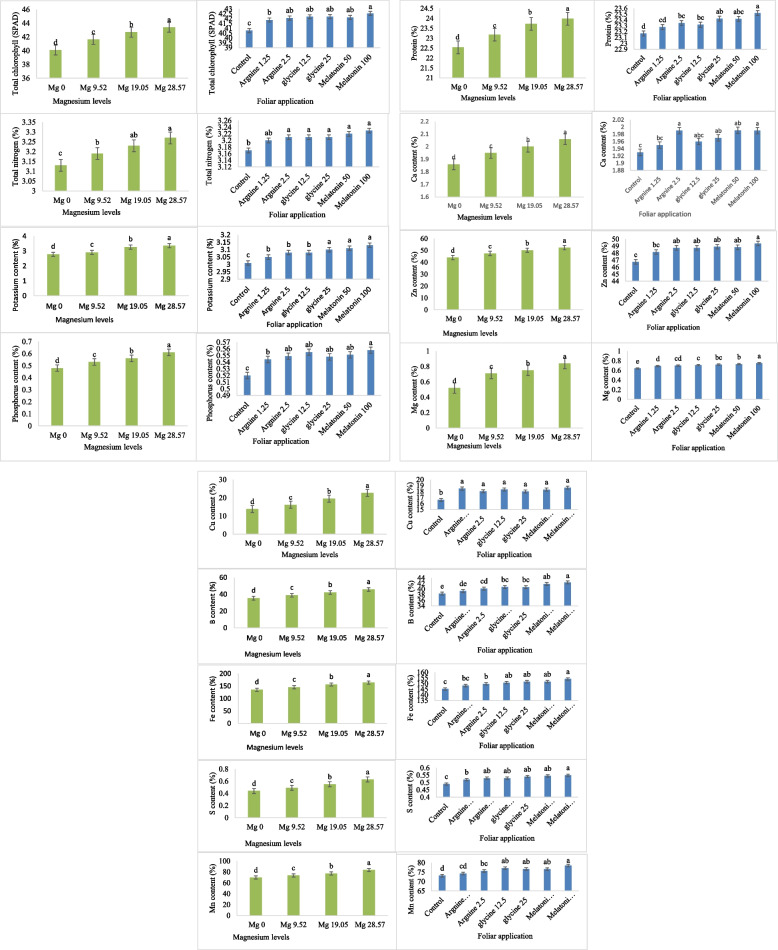
Effect of foliar application of arginine, glycine, control, melatonin, and Mg fertigation levels on total chlorophyll (SPAD), total nitrogen (%), potassium (%), phosphorus (%), crude protein (%), calcium (%), Mg (%), zinc (%), copper (%), iron (%), Sulfur (%), B (%), and Mn content (%). Different-letter bars indicate statistical significance at p ≤ 0.01. Error bars show the means'standard errors.

The physical and chemical parameters (total chlorophyll, total nitrogen, potassium content, phosphorus content, crude protein, Ca content, Mg content, Zn content, Cu content, Fe content, S content, B content and Mn content) were greatly influenced by Mg levels and foliar application treatments as shown by ANOVA analysis (Table 6). The interaction between Mg levels and foliar fertilization treatments was very significant on the following traits: K content, P content and Mg content, but it had no significant effect on the remainder of the physical and chemical parameters (Table 6). Depending on the foliar application treatments and magnesium levels, each characteristic reacted differently. Total chlorophyll (42.54 SPAD), total nitrogen (3.23%), potassium content (3.13%), phosphorus content (0.55%), protein (23.52%), Ca content (1.99%), Mg content (0.75%), Zn content (49.37%), Cu content (18.66%), Fe content (154.08%), S content (0.55%), B content (42.43%), and Mn content (78.5%) all had the highest levels of melatonin (100 µM) across the foliar application treatments (Fig. 8). Whereas the control was the lowest for all of these traits (total chlorophyll, total nitrogen, potassium content, phosphorus content, crude protein, Ca content, Mg content, Zn content, Cu content, Fe content, S content, B content and Mn content) where it recorded the following results (40.77 SPAD, 3.17%, 3.01%, 0.52%, 23.17%, 1.93%, 0.64%, 46.75%, 16.63%, 145.29%, 0.49%, 38.41% and 73.12%), respectively.

**Table 6:** Physical and chemical characteristics of common beans under various foliar spray treatments (arginine, glycine, melatonin, and control) and magnesium fertigation levels over two growing seasons are summarized in the analysis of variance summary table

Source of variation	Replication	Mg levels (A)	Foliar application treatments (B)	A*B
D.F	2	3	6	18
Chlorophyll (SPAD)	0.149	43.55^**^	3.81**	0.468^ ns^
Zn (%)	1.190	271.03**	8.69**	0.56^ ns^
B (%)	1.99	414.96**	23.09**	1.02^ ns^
S (%)	1.21e^−4^	0.136**	0.004**	1.87e-4^ ns^
Ca (%)	0.001	0.150**	0.007**	9.36e-4^ ns^
Mg (%)	4.21e-4	0.38**	0.014**	0.001**
K (%)	2.04e-4	1.61**	0.019**	0.001**
N (%)	9.77e-4	0.08**	0.004**	3.36e-4^ ns^
P (%	2.23e-4	0.065**	0.001**	4.21e-4**
Cu (%)	0.420	317.85**	5.565**	0.720^ ns^
Fe (%)	18.895	3327.725**	96.009**	7.963^ ns^
Mn (%)	1.151	729.579**	39.305**	5.088^ ns^
Protein (%)	0.016	8.447**	0.150**	0.015^ ns^

^**^: significance at 0.01 level

ns: Non-significant

In terms of the Mg treatments, the concentration (28.57 kg ha⁻^1^) of Mg was the highest across all traits, as shown by the following values: total chlorophyll was 43.41 SPAD, total nitrogen was 3.27%, potassium content was 3.36%, phosphorus content was 0.61%, the protein was 23.98%, Ca content was 2.06%, Mg content was 0.84%, Zn content was 52.38%, Cu content was 22.74%, Fe content was 164.09%, S content was 0.63%, B content was 45.75% and Mn content was 83.57% compared to the Mg concentration (0 kg ha⁻^1^) that produced its results (total chlorophyll was 40.10 SPAD, total nitrogen was 3.13%, potassium content was 2.78%, phosphorus content was 0.48,% protein content was 22.54%, Ca content was 1.86%, Mg content was 0.52%, Zn content was 44.02%, Cu content was 13.83%, Fe content was 135.21%, S content was 0.44%, B content was 35.38% and Mn content was 69.69%) (Fig. 8).

**Fig. 9:**
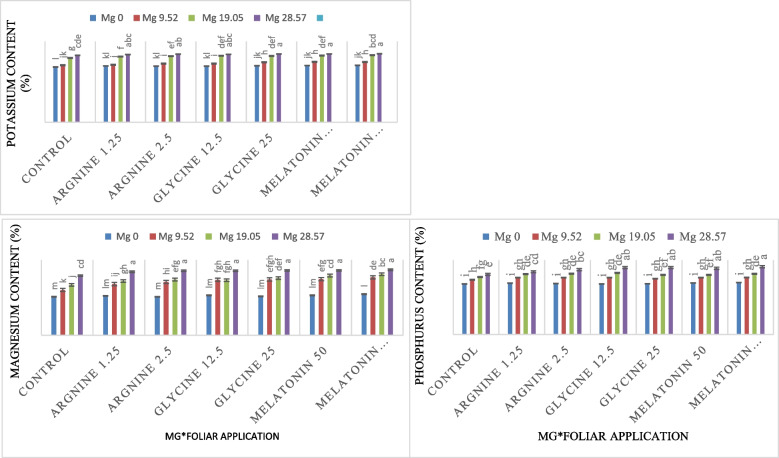
Impact of the interaction between Mg fertigation levels and foliar application of arginine, glycine, control and melatonin on potassium, Mg, and phosphorus content traits. Different-letter bars indicate statistical significance at *p* ≤ 0.01. Error bars show the means'standard errors

The relationship between Mg fertigation levels and foliar sprays of arginine, glycine, and melatonin had a substantial impact on the potassium content of common bean plants. With the highest values seen at the Mg 28.57 kg ha⁻^1^ level, rising Mg levels significantly improved potassium accumulation across all treatments. This impact was further enhanced by foliar administration of arginine and glycine, especially at greater dosages. Of all the treatments, melatonin 100 and magnesium 28.57 kg ha⁻^1^ resulted in the highest potassium concentration (3.39%). Higher potassium levels were also shown by melatonin 50 with Mg 28.57 kg ha⁻^1^ and glycine 25 with Mg 28.57 kg ha⁻^1^ (Fig. 9). However, the control plants had the lowest potassium content, particularly at Mg 0 kg ha⁻^1^ (2.73%), which suggests statistically lower values. The potassium content of the treatments: arginine 1.25, arginine 2.5, and glycine 12.5 was moderate. Concerning the phosphorus, manganese, and Mg content traits, were all at their highest with a concentration (100 µM) of melatonin under Mg level of 28.57 kg ha⁻^1^, on the other hand, the control treatment under Mg level of 0 kg ha⁻^1^had the lowest on these traits (Fig. 9).

**Fig. 10:**
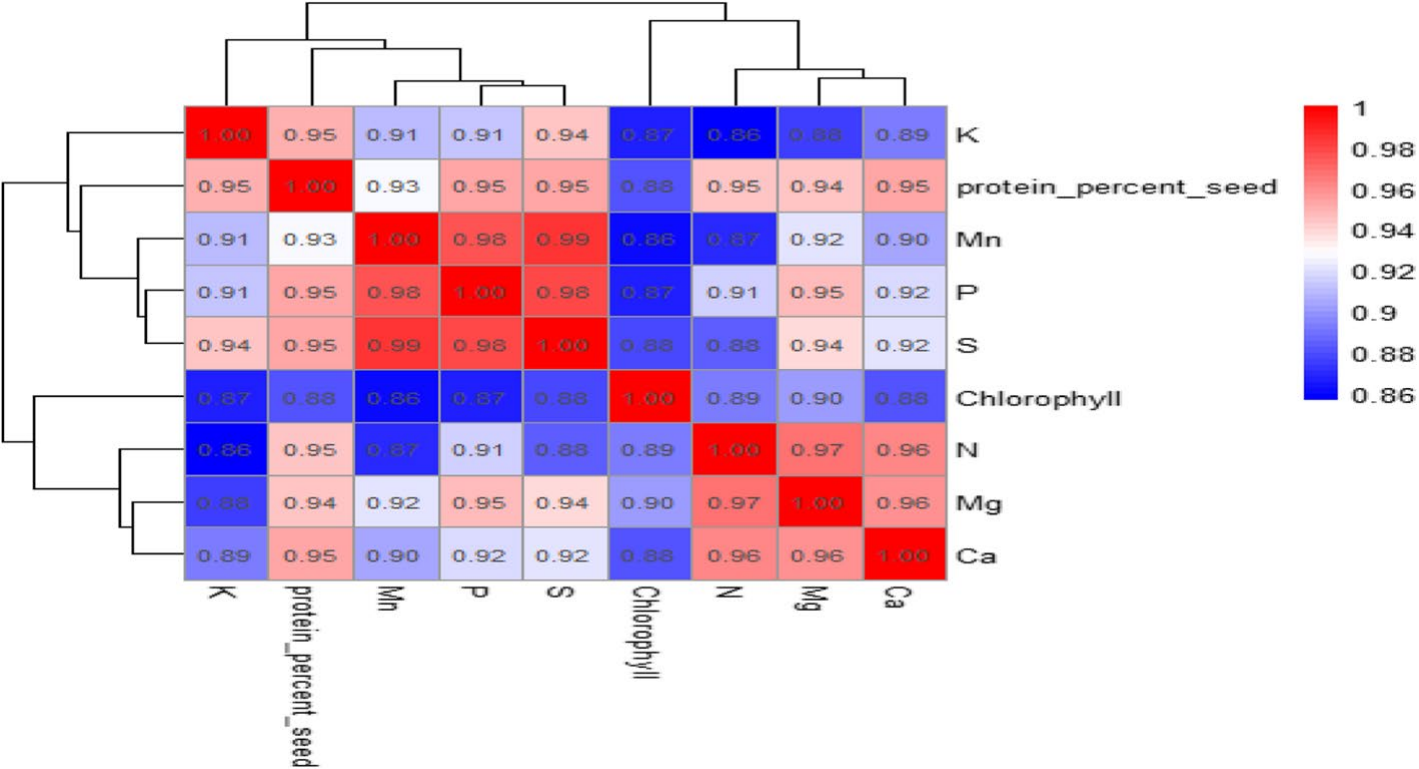
The correlations between the characteristics of the physical and chemical factors. The correlation coefficients between the chemical and physical characteristics are shown by the numbers in the figure. The color intensity indicates the strength and direction of the association. Strong positive correlations are indicated by red hues, whilst lesser correlations are indicated by blue colors. The strength and direction of the link are shown by the color scale

Since the majority of correlation coefficients are above 0.90, a significant correlation was seen across all attributes (Fig. 10). The range of the correlation coefficients was 0.86 to 0.98. The strongest correlation (*r* = 0.99) was seen between the sulfur and manganese contents. Additionally, there was a very good association (*r* = 0.98) between the amounts of manganese and sulfur and phosphorus. Additionally, there was a significant association (*r* = 0.97) between the amounts of magnesium and nitrogen (Fig. 10). Calcium content showed strong associations (*r* = 0.96) with Mg and nitrogen contents. Strong correlations were also found between sulfur content and both protein content (*r* = 0.95) and potassium content (*r* = 0.94). The nitrogen content also has a positive moderate correlation (*r* = 0.89) with total chlorophyll content. The smallest coefficient of association (*r* = 0.86) was found between manganese and chlorophyll contents and also between nitrogen and potassium contents (Fig. 10).

## Discussion

### The correlation between the agronomic traits

Seed yield per plant was highly positively correlated with 100 seed weight (r = 0.92). This indicates that seed weight increase is significantly associated with greater yield per plant. Seed yield per feddan was highly correlated with seed yield per plant (r = 0.95, *p* < 0.001), indicating that the productivity of the plant directly translates into yield on a larger scale [40]. The positive, strong correlation between the number of green pods per plant and dry pod weight per plant (r = 0.97) indicates the direct relationship between the number of green pods and the end yield of dry pods. It indicates that the plants with high numbers of green pods will have higher biomass accumulation, which will be translated into dry pod weight at maturity [43]. The strong correlation (r = 0.99) between plant leaf area and number of leaves indicates a developmental linkage that could enhance photosynthesis via expanded canopy surface, leading to increased biomass and yield [44, 45]. Plant height and leaf area showed a strong correlation (r = 0.96), reinforcing the idea that taller plants tend to support greater leaf expansion to optimize light capture and transpiration cooling, especially relevant under heat stress [46, 47]. Increased leaf surface may also support enhanced stomatal conductance and CO₂ fixation, promoting biomass synthesis [48]. Physiological correlations, such as the association (r = 0.89) between total chlorophyll and total nitrogen, highlight nutrient-dependent regulation of chlorophyll biosynthesis, which is key for photosynthetic efficiency [49].

### The role of melatonin in mitigating the effects of heat stress

One of the most important tasks facing modern agriculture is breeding crops to be more tolerant of heat stress [40, 41]. Scientists have extensively researched the possible role melatonin may play in enhancing plant development and regulation [50]. Melatonin is a multi-regulatory molecule that controls the gene expression related to abiotic stress resistance [51], redox reactions [52], and the development and growth of plants [53]. Studies such as Gu et al. (2024) [54] highlight diurnal variation in melatonin activity, suggesting complex regulation. The results of numerous studies on the function of phyto-melatonin are extremely important [55]. Plants that receive melatonin are more resistant to cold [56], high temperatures [57], salt [58], and drought [58]. Because of its important antioxidant role, melatonin helps plants better tolerate a variety of stressors, including heat, drought, exposure to heavy metals, salinity, cold temperatures, and fungal and viral infections [59]. Melatonin primarily reduces stress by strengthening the plant's antioxidant defense systems and assisting in the scavenging of reactive oxygen species (ROS) [60].

Research has demonstrated that when cucumber plants are stressed by cold and drought, smaller doses of melatonin (less than 10 mM) can enhance the germination of seeds and the formation of lateral roots [61]. Furthermore, melatonin has been demonstrated to improve plant stress tolerance in several ways. Melatonin, for instance, can suppress the metabolism of peroxidative promote the activity of antioxidants and activate genes linked to ion homeostasis, like AKT1 and NHX1 [62]. Our findings are supported by Imran et al. (2021) [63] and Oyemamiji et al. (2024) [64], who observed melatonin-enhanced tolerance of soybean and other species by maintaining photosynthetic performance, electrolyte balance, and antioxidant function. These physiological effects can likely be explained by melatonin's regulation of enzymatic ROS scavengers (e.g., SOD, CAT), osmolyte accumulation, and redox homeostasis [65].

### Impact of melatonin on the vegetative characters, seed parameters, pods parameters and yield of common bean

The control of cell walls by exogenous melatonin has received a lot of attention lately. In different herbaceous peonies (*Paeonia lactiflora*), melatonin content was positively associated with cell wall strength, indicating a relationship between melatonin and the cell wall [66]. To lessen the harmful effects, exogenous melatonin may control the biosynthesis of the cell wall, strengthen it, and improve its capacity to absorb ions. This would limit the amount of ions present in the cytoplasm [67]. Wei et al., (2015)[68] investigated how melatonin affected the development and growth of soybeans. The application of MT raised soybean seed size and plant height, according to the results. In addition, under abiotic stress, MT increased the number of seeds and pods, but not the weight of 100 seeds [69].

In addition, melatonin affects plant physiology by controlling vital functions such as the development of lateral roots and root primordia, carbon uptake, chlorophyll molecule breakdown, and the ascorbate–glutathione (AsA-GSH) cycle in stressed plants [70]. In our study, 100 µM melatonin maximized vegetative and reproductive traits with a dose-dependent protective effect against heat stress [71–73]. The findings can be explained based on improved chlorophyll preservation, nitrogen uptake, and protein biosynthesis [74], in agreement with Zhang et al. (2022) [75] and Chaurasia et al. (2023) [76], who found that melatonin enhanced sprout morphology and pod yield under salinity.

### Glycine's impact on common bean growth and yield under heat stress

The development and quality of common beans were assessed in lime soil using foliar and soil treatments with Fe-glycine chelate [77]. They found that the utilization of Fe-glycine treatments enhanced morphophysiological parameters. Compared to control, foliar application of Fe-glycine greatly increased leaf area. When plants were treated with Fe-glycine in both soil and foliar applications, there was a significant increase in pod yield, shoot dry weight, and levels of iron in leaves and pods [77]. According to our research, foliar glycine spraying positively impacted plant development compared to the control. This is explained by glycine's function in boosting enzyme activity, facilitating photosynthesis, and strengthening the plant's capacity to absorb nutrients [78]. By strengthening the plant's antioxidant defenses, glycine can help reduce stress and promote better growth overall. Glycine also may encourage the production of vital substances that improve growth performance, like proteins and chlorophyll [79]. Glycine may also act as a signaling molecule, triggering protein synthesis and chlorophyll accumulation [80, 81]. The antioxidant function of glycine, particularly via the synthesis of glutathione, is likely at the core of its heat-stress mitigation, protecting the photosystems and maintaining membrane integrity [73]. Glycine also maintains the reproductive characters by sustaining vegetative growth under stress.

### Impact of Arginine on common bean growth and productivity

The primary goal of much research on arginine's effects has been to make plants more resilient to abiotic stress. Plants treated with arginine were said to have higher yields and higher quality [82]. It's noteworthy to point out that the growth parameters under our study showed the strongest effect when arginine concentration was higher, which is consistent with the findings of Asadi-Sanam et al., (2018) [82]. They also observed that the higher concentration of arginine produced the most noticeable effect on the growth parameters examined. According to the results, applying arginine at a concentration of 2.5 mM improves several growth and yield metrics when compared to the control (distilled water). The 2.5 mM arginine treatment resulted in a higher plant height (59.24 cm) than the control (56.95 cm). This implies that arginine might encourage vertical growth. As an amino acid that is essential to plant metabolism, arginine serves as a precursor for polyamines (such as putrescine and spermidine), which are known to affect cell division as determined by Aroca et al., (2013) [83]. As a result, increased cell division and elongation processes may be the cause of elongation height. Additionally, 100 seeds treated with arginine weighed more than the control. This might be the result of enhanced metabolic activity, higher nutrient absorption, and enhanced general plant health brought on by arginine [84]. Synthesis involves amino acids, which may improve the quality and development of seeds. Additionally, the amount of chlorophyll in the plants treated with arginine increased in our investigation. This rise points to increased photosynthetic efficiency, which could be connected to greater plant growth and general health [85]. By taking part in nitrogen digestion and stimulating the activity of enzymes involved in chlorophyll formation, amino acids such as arginine can affect the pathways involved in chlorophyll biosynthesis [86]. Our study supports findings by Yeboah et al. (2021) [87], showing arginine increased vegetative and reproductive performance via improved nitrogen metabolism and osmotic balance. These physiological effects, including enhanced chlorophyll biosynthesis, likely stem from its influence on nitrate reductase and enzyme-mediated synthesis of metabolic intermediates [88].

### Magnesium's impact on common bean growth and yield under hot conditions

The absence of Mg in many weathered soils is concerning because it is essential for photosynthesis, net assimilation, and relative growth and yield [88]. Magnesium's ability to function as a cofactor in practically all phosphorylative enzymes involved in plant metabolism is its primary characteristic [89]. According to Sultana et al., (2016)[90], plants lacking Mg exhibit a substantial decrease in the production of carbohydrates, proteins, and chlorophyll and this is completely consistent with what was found in this study. For crops to grow and yield as much as possible, a quick and steady availability of Mg is required. This is especially crucial for common beans because of their limited root system [89]. According to our findings, when compared to the control (0 kg kg ha⁻^1^), the greatest Mg content (12 kg ha⁻^1^) significantly enhanced all measured features, indicating that Mg is essential for improving nutrient absorption and overall plant metabolism [91]. The observed rise in total chlorophyll concentration was probably caused by Mg's function as a key atom in the chlorophyll molecule [92]. Magnesium may also improve carbon absorption and photosynthetic efficiency, which would increase protein synthesis and nutrition buildup [93].

The overall chlorophyll concentration rose from 40.10 to 43.41 after Mg therapy. Given that Mg is an essential part of the chlorophyll molecule, this implies that Mg has a positive effect on photosynthesis. The percentage of protein rose from 22.54% to 23.98%. This increase in protein concentration is probably explained by Mg's role in protein synthesis and general metabolic activity. These increases in micronutrient content imply that Mg improves plant uptake and utilization of micronutrients, which are essential for several physiological processes, such as stress resistance, photosynthesis, and enzyme activation [89].

### The relationship between Mg, high temperatures, and foliar applications

Magnesium is a central actor in plant physiology, particularly as a component of the chlorophyll molecule, which directly influences photosynthetic efficiency and overall plant health [91]. In this study, Mg application significantly improved vegetative traits, especially at the highest level (28.57 kg ha⁻^1^), vindicating its pivotal function in promoting chlorophyll content and carbon fixation, particularly under stress such as high temperature. These findings are consistent with previous evidence that included magnesium as a necessary ingredient in structural ribosome stabilization, photochemical enzyme activation, and heat-stressed ATP production maintenance [84]. Notably, our research revealed a clear interaction between foliar treatment applied and Mg, with pronounced synergisms present. Specifically, concomitant use of Mg and foliar sprays, especially melatonin, created spectacular enhancements in plant growth, potassium, phosphorus, and magnesium levels. This suggests a synergism, where magnesium optimizes the effectiveness of chemicals applied through the foliage to facilitate nutrient uptake and stress resistance. While melatonin was extremely potent, other foliar sprays also enhanced the expression of the trait, although to varying levels. For instance, ascorbic acid and salicylic acid treatments also promoted plant performance but exerted greater impacts at moderate magnesium levels, indicating potential differential mechanisms of action among the treatments.

[85]. The rise in potassium composition observed with combined treatments is quite intriguing since potassium has been found to regulate stomatal movement, maintain cellular turgor, and buffer oxidative stress, all of which are crucial during high temperature stress [93]. The enhanced deposition of potassium in our study further validates the efficacy of magnesium-foliar synergy in promoting physiological balance and stress adaptation [87]. The physiological improvements observed, e.g., greater chlorophyll content, greater leaf area, and greater accumulation of nutrients, all have a direct bearing on greater yield potential. Greater chlorophyll levels enhance light capture and energy production, and greater leaf area provides a greater surface area for photosynthesis, both of which are conducive to the production of more assimilates needed for reproductive growth [89]. In addition, elevated potassium levels guarantee turgor and stomatal conductance retention, which helps in carbon fixation and transport of carbohydrates to the developing pods and seeds. All these effects together lead to enhanced flowering, podding, and eventually greater yield components such as the number of pods, seed weight, and total yield [86]. In summary, our findings demonstrate that the synergy of Mg fertilization with foliar applications, particularly melatonin, can effectively enhance plant performance under stress conditions. Our results suggest that the integration of Mg with certain foliar applications could be an effective agronomic strategy to increase crop tolerance to hot environments.

### Limitations and future research directions

Though the study demonstrates robust evidence for the effectiveness of exogenous melatonin, glycine, arginine, and magnesium against heat stress, it was conducted in a single field over one growth season. Environmental interactions such as soil heterogeneity, long-term climate change, and cultivar-specific reaction were not completely resolved. Moreover, the precise molecular mechanisms such as transcription factor regulation and hormone interaction remain to be understood. More research needs to involve transcriptomic and metabolomic profiling, multi-location scale-up trials, and analyze gene expression dynamics in order to establish mechanisms of action across different agro-climatic zones.

## Conclusion

According to the findings, arginine, glycine, and melatonin, particularly Melatonin 100 µM, have a significant favorable impact on the common bean's vegetative traits and grain yield components, particularly when heat stress is present. This seems to strengthen the plant's resistance to stress, which could lead to increased growth and yield. We propose that the common bean's heat tolerance during the sprouting stage may be improved by the interaction of the cell walls with external melatonin. In particular, it was discovered that applying 28.57 kg per hectare of magnesium (Mg) produced the best yield, underscoring the importance of this mineral in improving plant performance under challenging circumstances like heat stress. According to these results, using Mg in combination with these substances may be a viable way to lessen the negative effects of heat stress on common beans, enhancing plant health and production under trying environmental circumstances. Future research can examine this aspect to determine the maximum amount of time that common beans can withstand abiotic stressors, especially heat stress.

## Data Availability

No datasets were generated or analysed during the current study.

## Declarations

**Ethics approval and consent to participate.**

Not applicable.
